# p53 aberrations in low grade endometrioid carcinoma of the endometrium with nodal metastases: possible insights on pathogenesis discerned from immunohistochemistry

**DOI:** 10.1186/s13000-017-0668-6

**Published:** 2017-11-14

**Authors:** Oluwole Fadare, Vinita Parkash

**Affiliations:** 1 Department of Pathology, University of California San Diego Health, 9300 Campus Point Drive, Suite 1-200, MC 7723, La Jolla, CA 92037 USA; 2 Department of Pathology, Yale School of Medicine, New Haven, CT USA

**Keywords:** Endometrioid carcinoma, Uterus, p53-aberrant, Driver, p53-wild-type

## Abstract

**Background:**

*TP53* mutations are rarely identified in low grade endometrioid carcinoma of the endometrium, and their pathogenic significance in such tumors is evidenced by the fact that *TP53* aberrations have been associated with reduced recurrence-free survival in this subset of tumors. However, *TP53* aberrations may not always represent a driving molecular event in a given endometrial cancer with a mutation. In this case study, the immunophenotype of a distinctive low grade endometrioid adenocarcinoma with an unusual pattern of lymph node metastases is used to explore the possible roles for underlying *TP53*-related molecular events in its pathogenesis.

**Case presentation:**

A low grade endometrioid carcinoma, 9 cm in greatest dimension, with 35% invasion of the myometrial wall thickness, focal lymphovascular invasion, and metastases to 2 of 16 pelvic lymph nodes, was diagnosed in a 52-year-old woman. The endometrial tumor showed a p53-mutation (aberrant)-type immunohistochemical pattern in 40% of the tumor, but the rest of the tumor, as well as the foci of myometrial and lymphovascular invasion, were p53-wild type. Both lymph nodes with metastatic disease showed a distinct biphasic pattern, comprised of both p53-wild type and p53-aberrant areas in tumoral foci that were spatially apposed but not intermixed. Most p53-aberrant areas (at both the lymph nodes and the endometrium) showed a higher mitotic index and increased atypia as compared to the p53-wild type areas; both showed squamous differentiation. The p53-aberrant areas at both locations were also p16-diffusely positive, vimentin-positive, and estrogen/progesterone receptor-positive, whereas the p53-wild type areas showed an identical immunophenotype with the exception of being p16-mosaic positive. All components of the tumor at both the primary and metastatic sites showed loss of MSH2 and MSH6 and retained MLH/PMS2 expression.

**Conclusions:**

The presence of p53-mutant and wild-type areas in multiple lymph nodes, coupled with the absence of a p53-aberrant immunophenotype in the myometrium-invasive or lymphovascular-invasive portions of the tumor, argues against the possibility that the *TP53* mutation in this tumor is a driving event in its pathogenesis, at least regarding the metastatic process. This case illustrates how routine immunohistochemistry can provide important insights into underlying molecular events in cancers, exemplifies an uncommon co-existence of DNA mismatch repair protein deficiency and p53-aberrant immunophenotype in low-grade endometrioid carcinoma, illustrates morphologic differences between p53-aberrant and p53-wild type areas within in the same tumor, and is an exemplar of the emerging theory that lymph node metastases of endometrial cancer may be comprised of different subclones of the primary tumor.

## Background

The *TP53* gene, which is located on 17p13.1 [1], encodes the p53 protein and is one of the most frequently mutated genes in human cancers [2, 3]. p53 occupies a central position in a vast, likely integrated but incompletely understood network of cellular signaling that essentially maintains the “genomic health” [3, 4]. Accordingly, inactivation of p53 may result in a cellular environment that is conducive to or permissive of oncogenesis [3, 4]. Inactivation of p53 may occur through a variety of mechanisms, including mutation of the p53 gene, binding to viral proteins, expansion of its negative regulators, or other alterations of genes and proteins that are directly or indirectly involved in p53-mediated signaling [4, 5]. The p53 content of cells is generally kept at low levels through an ubiquitin-effectuated proteolytic process that is primarily mediated by the protein MDM2, itself a p53 target, thereby creating a negative feedback loop [4–6]. Mutant forms of p53, in contrast, are stable and accumulate to high levels intracellularly. This has traditionally been attributed to the inability of the p53 mutant protein to optimally transactivate its negative regulator (MDM2), but a comprehensive picture of all contributing factors to the process remain unclear [4–6]. p53 function is inactivated in most human cancers through missense or nonsense/frameshift mutations of the gene [7], which respectively correspond to the protein “overexpression” and “null” patterns of aberrant (i.e. non wild type) p53 immunoreactivity [8].

Among the histotypes of endometrial carcinoma, p53 aberrations represent the most frequently identified recurring molecular event in serous carcinomas [9, 10] and carcinosarcomas [11, 12] but have also been identified in significant subsets of high grade endometrioid [13], clear cell [14] and dedifferentiated/undifferentiated carcinomas [15, 16]. Numerous reports have found p53 aberrations to be a significantly negative prognostic factor in endometrial carcinomas in general, although how independent this significance is of the necessarily dependent variables of tumor histotype and tumor grade has been a subject of some debate [17–22]. p53 aberrations are very rarely identified in low grade endometrioid carcinomas and are occasionally identified in intermediate-grade carcinomas. In the TCGA (The Cancer Genome Atlas) dataset, 0 and 11.8% of grades 1 and 2 endometrioid carcinomas had a *TP53* mutation [9]. Recently, Kurnit et al. examined 125 cases of grades 1 and 2, stage 1 and II endometrioid carcinomas by next generation sequencing, and found *TP53* mutations, which were present in 9% of cases, to be associated with worse recurrence-free survival on multivariate analysis [22]. This suggests that the “acquisition” of a *TP53* mutation in a low-stage, low-grade endometrioid carcinoma is of adverse prognostic significance and is accordingly a significant event in its pathogenesis.

Only 7% of endometrial carcinomas show a DNA mismatch repair protein (MMR) deficiency that is unrelated to MLH1 promoter hypermethylation [23], and as was previously indicated, *TP53* mutations are uncommon in low grade endometrioid carcinoma [9]. The combination, i.e. *TP53* mutations in a low grade endometrioid carcinoma with MMR deficiency unrelated to promoter hypermethylation, is distinctly uncommon. In this report, the authors describe one such case - a low grade endometrioid carcinoma of the endometrium showing a p53-aberrant immunophenotype in one portion of the tumor, MSH2 and MSH6 loss in all components, and a distinctive pattern of lymph node metastases in multiple lymph nodes comprised of both the p53 wild-type and p53-aberrant components. The unique aspects of this case are used to explore the role of *TP53* mutation in the case, its implications on the subclone theory of tumor metastases, and how immunohistochemistry may potentially provide valuable insights into these events

## Case presentation

**Fig. 1:**
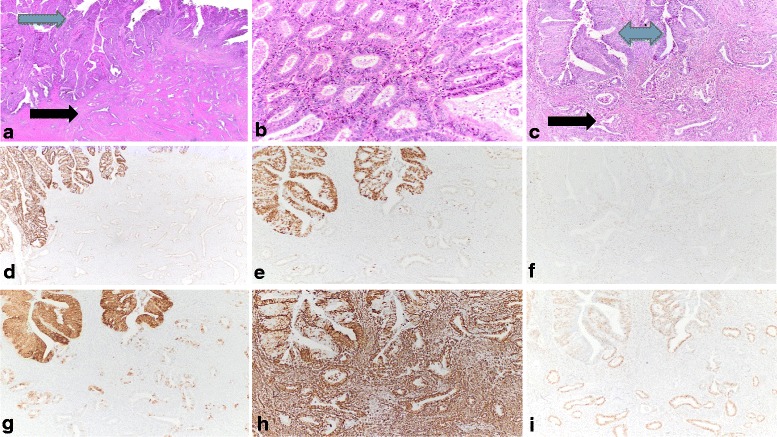
Morphologic and immunophenotypic features of tumor in the uterus: **a** Focus of myometrial invasion (black arrow) with overlying endometrial tumor (blue arrow) (H and E, original magnification: 2 x). **b** The p53-wild type areas of the tumor (H and E, original magnification: 40X). **c** Transitional areas between myoinvasive glands (black arrow) and overlying, non-myoinvasive parts of tumor (blue arrow) (H and E, original magnification: 40X). **d** and **e** p53 immunohistochemistry showing wild-type staining pattern in the myoinvasive glands and aberrant staining pattern in the overlying, non-myoinvasive parts of tumor (original magnifications: 1D: 2X; 1E: 10X). **f** Loss of MSH2 in both components of the tumor (original magnification: 10×). **g** p16 immunohistochemistry showing mosaic staining pattern in the myoinvasive glands and diffuse staining pattern in the overlying, non-myoinvasive parts of tumor (original magnification: 10X). **h** Vimentin expression in both components of the tumor (original magnification: 10×). **i** Estrogen receptor expression in both components of the tumor (original magnification: 10×)

**Fig. 2:**
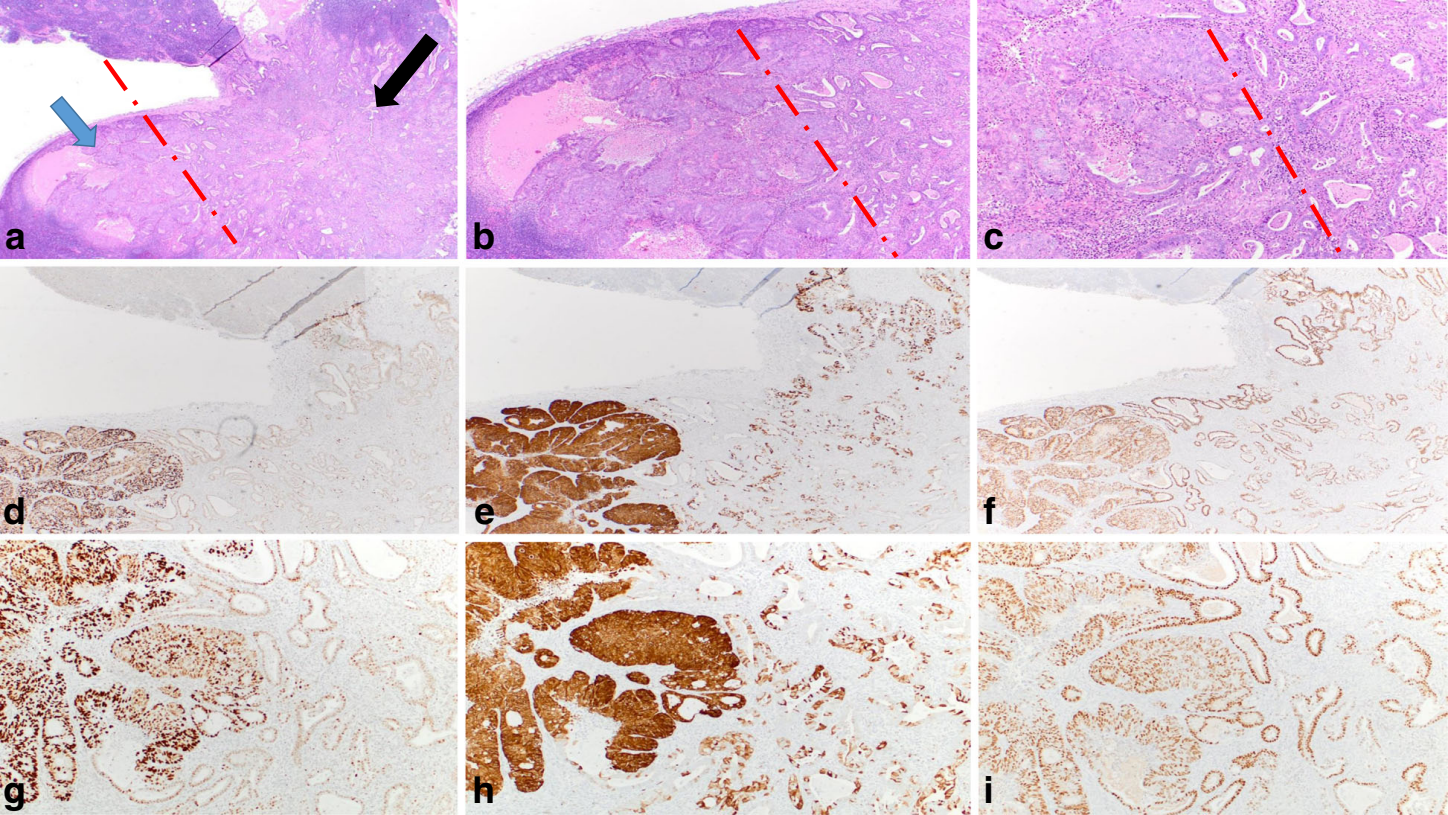
Morphologic and immunophenotypic features of tumor in one lymph node. **a**-**c** The 2 components of the tumor (ie areas that were ultimately classified as p53 wild-type (black arrow) and p53-aberrant (gray arrow) were apposed but not intermixed. An red line approximately separates the 2 areas. [H and E, original magnification: 2 x (1A); 4× (1B); 10× (1C)]. **d**, **g** p53 immunohistochemistry, showing different patterns of expression in both areas of the tumor [original magnifications: 2 x (1D); 10× (1G)]. **e**, **h** p16 immunohistochemistry, showing different patterns of expression in both areas of the tumor [original magnifications: 2 x (1E); 10× (1H)]. **f**, **i** Progesterone receptor immunohistochemistry showing similar patterns of expression in both components [original magnifications: 2 x (1E); 10× (1H)]

A 52-year-old woman, gravida-3, para-3, 3 years post-menopausal, presented with post-menopausal bleeding of “several weeks” duration. She ultimately underwent a biopsy on which a diagnosis of endometrioid adenocarcinoma, FIGO grade 1 was rendered. Imaging showed a left adnexal mass whose features were equivocal regarding benignancy. A decision was made to perform a total hysterectomy and bilateral salpingo-oophorectomy. An intraoperative pathologic assessment was performed, which showed the adnexal mass to be benign but the endometrial mass to be of large volume (a 9 × 6 cm soft polypoid mass that occupied the entirety of the uterine cavity) with myometrial invasion and lower uterine segment involvement. A bilateral pelvic lymphadenectomy was also performed. Microscopic examination of the permanent sections of the case showed a grade 1 endometrioid carcinoma of the endometrium, with 35% invasion of the myometrial wall thickness, focal lymphovascular invasion, and metastases to 2 of 16 pelvic lymph nodes (Figs. 1 and 2). Approximately 40% of the tumor was comprised of columnar glands showing more nuclear stratification, more nuclear enlargement, and more prominent nucleolomegaly than the background glands (atypical areas). These areas also showed comparatively increased mitotic indices (average 17 MF/10 HPF) than the background glands (average 9 MF/10 HPF), from which they were spatially distinct. Immunohistochemically, the atypical areas showed a p53 aberrant immunophenotype, characterized by diffuse and marked nuclear positivity for p53 in more than 90% of lesional nuclei. The p53-aberrant areas were also p16-diffusely positive, vimentin-positive, Napsin A-negative, estrogen receptor positive, and progesterone receptor-positive (Fig. 1). The remainder of the tumor (60% of tumoral volume) displayed a p53-wild type immunophenotype, and were p16-mosaic positive, vimentin-positive, Napsin A-negative, estrogen receptor positive, and progesterone receptor-positive. As such, the p53-aberrant and p53-wild type areas showed an identical immunophenotype with the exception of the latter being p16-mosaic positive. Foci of lymphovascular invasion and myometrial invasion showed a p53-wild type immunophenotype and were identical in immunophenotype to the other p53-wild type areas within the tumor. Areas of background hyperplasia showed a p53 wild type immunophenotype. The endometrioid carcinoma at its primary site showed minor squamous differentiation, and no solid components in both the p53-wild type and p53-aberrant areas.

The 2 lymph nodes with metastatic disease each showed a distinct biphasic pattern, comprised of both p53-wild type and p53-aberrant areas in foci that were spatially apposed but not intermixed. The p53-aberrant areas were identical in morphology and immunophenotype to the “atypical areas” of the endometrial tumor described above. The p53-wild type tumoral areas were comprised of glands with less columnar configuration and more non-specific cytoplasmic clarity. However, they showed foci of squamous differentiation and were otherwise immunophenotypically identical to the p53-wild type areas within the endometrial tumor (Fig. 2). All components of the tumor at both the primary and metastatic sites showed loss of MSH2 and MSH6 with retained expression of MLH1 and PMS2. The patient declined germ-line testing, and underwent adjuvant chemotherapy. The case is too recent for meaningful follow-up.

All immunohistochemical studies were performed on 4 μ-thick, unstained slides of formalin-fixed, paraffin-embedded tissue sections using the Ventana Benchmark automation and the Ultra View detection kit (Ventana Medical Systems, Tucson, AZ) and the following primary antibodies: Estrogen receptor (ER; Clone SP1; prediluted, Ventana), Progesterone receptor (PR; clone IE2; prediluted; Ventana), p53 (clone DO-7; dilution 1:40; Ventana), Napsin-A (polyclonal, prediluted; Cell Marque, Rocklin, CA), Vimentin (clone V9, dilution 1: 1000, Ventana), p16 (Clone JC8, prediluted, Santa-Cruz, Dallas, TX), MLH1 (clone G168–15, prediluted, Biocare Medical, Concord, CA), PMS2 (Clone A16–4; dilution 1:25; BD Biosciences, San Jose, CA), MSH2 (clone FE11, prediluted, Biocare Medical), and MSH6 (clone BC/44, prediluted, Biocare Medical).

## Discussion

Approximately 8% of the 65 hypermutated (microsatellite unstable) endometrial carcinomas in the TCGA data set showed a *TP53* mutation [9]. The grade distribution for those *TP53*-mutant, hypermutated cases were not outlined [9]. However, in one comparable analysis of 319 cases that sought to recreate the molecular subgroups of the TCGA study, only 8 (2.5%) of 319 cases concurrently displayed a *TP53* mutation and a DNA MMR protein deficiency, and all 8 cases were high grade carcinomas [24]. A case of a low-grade endometrioid carcinoma of the endometrium showing a p53-aberrant immunophenotype in one portion of the tumor, MSH2 and MSH6 loss in all components, and a distinctive pattern of lymph node metastases, is described herein. It is the authors’ experience that this combination is extraordinary uncommon.

DNA mismatch repair proteins recognize and correct errors that may arise during DNA replication and recombination, and inactivation of DNA mismatch repair can increase spontaneous mutation rates, which in turn increases the likelihood of oncogenesis [25]. Similarly, among a myriad of other cellular functions, *TP53* is a tumor suppressor gene that prevents the propagation of potentially oncogenic events by inducing apoptosis [1–7]. Therefore, a loss of function in both systems may theoretically have a synergistic effect. Indeed, in vitro analyses of colon cancer cell lines has shown that loss of function of both systems synergistically increases the frequency of mutations, promotes genomic instability under stress, and increases chemoresistance [26–28]. Other in vitro evidence suggest the existence of some interaction between the 2 systems, but a clear picture is lacking [29–32].

Somatic mutations in cancer genomes have traditionally been categorized into 2 major groups based on the consequences of those mutations on oncogenesis and/or cancer evolution: “Driver” mutations result in a selective growth advantage to cells that harbor them [33, 34]. Cells with driver mutations were selected for at some point in the evolution of the cancer, and are often implicated in the causality and maintenance of the cancer [33]. As such, they are identified at a relatively high frequency in a cancer of a particular type [35]. “Passenger” mutations, which greatly outnumber driver mutations, have traditionally been thought to not confer a growth advantage and to have a limited role in oncogenesis [33]. Recently, an additional “mini-driver” model has been proposed, in which some somatic mutations are neither passengers nor drivers, but are “mini-drivers”, each with “relatively weak tumor-promoting effects”, but which may cumulatively display major tumor-driving tumorigenic effects under the right conditions [36]. Other recent proposals include the “latent driver” model in which some mutations generally behave as passengers but can drive cancer development when coupled with other mutations [37], and a model in which passenger mutations are not simply neutral events, but which may have cumulative effects that actually slow or prevent tumor progression [38, 39].

The morphologic and immunophenotypic findings in the current tumor are of interest in deciphering whether the p53 mutation that was apparently acquired in one portion of the tumor was a driving or passenger event in lymph node metastases. As was previously noted, *TP53* mutations in low and intermediate grade endometrioid carcinomas have been associated with reduced survival [22], and studies of endometrial cancer cell lines have showed that *TP53* mutation increases invasion and migration, and therefore likely promote metastases [40]. However, in the current case, it is unlikely that *TP53* mutation was the primary driving event. Most −60%- of the tumor was p53-wild type, including foci of background hyperplasia, which suggests that the p53-aberrant areas were a manifestation of a subclone of p53-mutated tumor within this background. The areas of the tumor that displayed “aggressive” features (i.e. myometrial invasion and lymphovascular invasion) all showed a p53-wild-type immunophenotype, which argues against the possibility that the p53-aberrant clone was particularly aggressive. More importantly, the findings in the lymph nodes argue against the possibility. If there had been a clonal expansion of an aggressive *TP53*-mutant clone in the endometrial cancer, the lymph node metastases would be expected to be p53-aberrant in their entirety. In contrast, the presence of multiple lymph nodes with p53-wild type tumor concurrent with their p53-mutant counterparts suggests that the *TP53* aberration in this case may function no more than as a mini-driver, possibly synergizing with other molecular events, but not being the primary driving force in the metastatic process.

This case may also have some implications regarding the larger “subclone” theory of metastases [41]. This theory proffers that a given cancer is composed of subpopulations (subclones) of cells that are heterogeneous regarding a variety of molecular properties, including the potential for metastasis, and that subclones with metastatic potential can emerge at various points in the lifecycle of a tumor [41]. One recent study of colorectal cancer in which high-confidence phylogenetic trees were constructed using hypermutable DNA found that in 65% of cases, metastases arose from independent subclones of the primary tumor [42]. An earlier study, also on colorectal cancer, had reported that individual node metastasis are comprised of multiple sub-clones from the primary tumor [43]. In the current case, the primary tumors and their metastases indeed showed an identical overall immunophenotype. However, the two tumor-positive lymph nodes each seemed to harbor 2 clones of the same tumor, with each node showing p53-wild type areas and p53-aberrant areas in different areas of a contiguous nodal deposit. One potential explanation is that the *TP53* mutation in the lymph node metastases was newly acquired there, and is unrelated to the *TP53* mutation in the endometrial tumor. In support of this possibility is the finding that about one-half of mutations that are present in endometrial cancer metastases are absent in their matched primary tumors [44]. Arguing against this possibility is the fact that in the present case, metastases in two different lymph nodes showed this “bi-clonal” morphology, since it is highly unlikely that metastatic deposits in 2 separate lymph nodes each acquired an unrelated *TP53* mutation. Overall, the subclone theory appears to be more probable.

Irrespective of how the nodal tumors acquired a *TP53* mutation, the pattern of metastases arguably bolsters the point that the *TP53* mutation in this case was not the driving event, at least with regard to the promotion of lymph node metastases. As was previously noted, a clonal expansion of an aggressive *TP53*-mutant clone in the endometrial cancer should result in lymph node metastases that are p53-aberrant in their entirety. In contrast, *TP53*-aberration as a passenger mutation may theoretically be unrelated to the metastatic process in the case. The critical molecular event driving metastases appeared to be, at minimum, incompletely dependent on *TP53*. It is unclear whether the driving event in this case is loss of MSH2/MSH6. In one recent study from the ProMisE (Proactive Molecular Risk Classifier for Endometrial Cancer) group, patients in the MMR-deficient subgroup (defined as those displaying loss of PMS2 and MSH6) had relatively poor outcomes [24], although in general, the prognostic significance of DNA MMR protein deficiency in endometrial cancer is still the subject of debate [45, 46].

It is perhaps expected that tumors with a high mutational burden, including ultramutated cancers (typically those harboring a POLE exonuclease domain mutation) and hypermutated cancers (typically those that are microsatellite unstable) would also harbor the most commonly altered cancer gene, *TP53*, with a significant frequency. The *POLE* mutation status of the present case is unknown. Nonetheless, the ongoing challenge in cancers in general is to identify the most significant alterations. With the increasing availability of genomic information in cancers, distinguishing between passenger mutations with limited clinical consequence, and driver mutations that would likely affect outcome takes on added importance. We hypothesize that in endometrial endometrioid carcinomas, the role of *TP53*, much like the gene itself, is variable, driving pathogenesis in some instances, being a passenger in others, and serving in others in a myriad of “intermediate” roles in another subset.

**Fig. 3:**
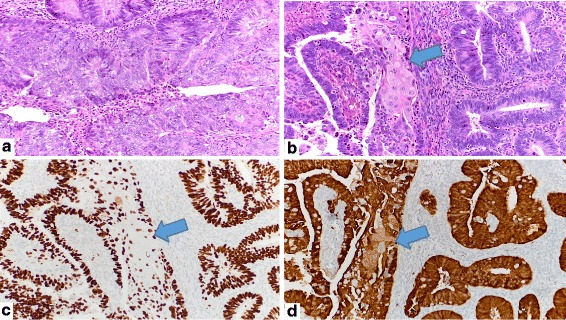
Morphologic and immunophenotypic features of the p53-aberrant areas of tumor at its primary site (arrows indicate focus of squamous differentiation, consistent with an endometrioid histotype). **a**, **b** Morphologic features, showing increased atypia and mitotic activity in a p53-aberrant area of the tumor, compared to the p53-wild type areas as illustrated in Fig. 1b [H and E, original magnification: 40 x]. **c** p53-aberrant immunophenotype. [original magnifications: ×40]. **d**, 1H: p16-diffusely positive immunophenotype. [original magnifications: ×40]

The proportion of low grade endometrioid carcinomas that display a *TP53* mutation is very low [9, 22], and it is even more uncommon in our experience for a low grade endometrioid carcinoma to show a p53-aberrant immunophenotype in only one portion of the tumor. This offered a rare opportunity to study within the same case the morphologic and immunophenotypic differences between the p53-aberrant and p53-wild type areas. At both their primary and metastatic sites, we found that the p53-aberrant areas were more mitotically active than the p53 wild-type areas and showed more atypia, as evidence by more nuclear stratification, more nuclear enlargement, and more prominent nucleolomegaly (Fig. 3). However, we did not think that the changes were sufficiently distinctive to allow the confident delineation of cancers that are likely to be p53-aberrant by their morphologic features alone.

## Conclusions

This case illustrates how routine immunohistochemistry can provide important insights into underlying molecular events in cancers, exemplifies an uncommon co-existence of a DNA mismatch repair protein deficiency and a p53-aberrant immunophenotype in low-grade endometrioid carcinoma, illustrates morphologic differences between p53-aberrant and p53-wild type areas in the same tumor, and is exemplary of the emerging theory that lymph node metastases may be comprised of different subclones of the primary tumor.

## Abbreviations

DNA: Deoxyribonucleic acid
HPF: high power fields
MDM2: Mouse double minute 2 homolog
MF: mitotic figures
MLH1: MutL homolog 1
MSH2: MutS protein homolog 2
MSH6: MutS protein homolog 6
PMS2: PMS1 Homolog 2
POLE: DNA Polymerase Epsilon
TCGA: The Cancer Genome Atlas
TP53: Tumor protein p53
